# Indents

**Published:** 1910-01-15

**Authors:** 


### INDENTS.

☞**Brief Articles of Technical or Scientific Value will receive notice and comment. Contributions invited.**

*Paraffine Root Filling.*

| | |
|---|---|
| Melted paraffin | ʒvii |
| *Paraoorm* | ʒi |
| Zinc Oxide | ʒiv |

—*The Journal.*

*The Scourge of the North.* This name is appropriately bestowed by *The British Medical Journal* (London, November 20) upon what is generally called the "common cold"—a distressing malady with which medical science has not dealt adequately. The trouble is, *The Journal* points out, that this disease is not one, but many, its symptoms being due to germs of various kinds. In particular, the writer condemns the idea that colds may be avoided by "hardening" one-self. He says:

"The common cold is almost as great a scourge to the inhabitants of Northern Europe and America as the touch of fever to the tropical resident. The multitude of remedies, domestic, medical, and proprietary, ranging from blackberry tea and a tallow candle to opium and smelling salts and nasal sprays, is a proof that no really effectual remedy is known. Dr. Benham, in his recent interesting report to the Science Committee of the British Medical Association holds out some hope that a vaccine may cut short an attack or help to make it bearable; but unfortunately the microbe is not always the same, so that it would be necessary first to discover the name of the enemy or to use a mixed vaccine. On the sound principle that prevention is better than cure, many people have endeavored to harden themselves against catching cold, and the hatless brigade claim that they are immune to colds in the head, or at least much less susceptible than the generality. The use of the morning cold tub is praised for the same reason, and there are people who regard the wearing of a greatcoat in winter as a direct inducement to the microbes to work their evil will. There is no reason why robust adults should not follow their beliefs and inclinations in this matter, but for adults who are not robust and for children the hardening process is not free from risk."

Upon this point the writer quotes Dr. A. Kuhn who has recently published a paper on the prevention of colds. This authority seems to think that a cold is not usually caught from another person, but that a chill affords to microbes already in the nose, throat, or mouth, conditions favorable to their activity, by altering the cutaneous circulation. To quote further:

"In dealing with plans for hardening the body to resist the noxious influences of cold, he utters a word of warning against exaggeration. The body must be kept warm, and this is particularly true in the case of children. He does not approve of coldwater hardening for very young children, but advises the gradual resort to cold bathing at a later age. Throughout his articles he preaches moderation, and advises the use of cold water, fresh air, exercise in the open air, and so on, in such measure as the individual can easily tolerate without feeling a sensation of coldness or discomfort."

